# ERK1/2 signaling regulates the immune microenvironment and macrophage recruitment in glioblastoma

**DOI:** 10.1042/BSR20191433

**Published:** 2019-09-13

**Authors:** Claire Lailler, Christophe Louandre, Mony Chenda Morisse, Thomas Lhossein, Corinne Godin, Marine Lottin, Jean-Marc Constans, Bruno Chauffert, Antoine Galmiche, Zuzana Saidak

**Affiliations:** 1 Equipe CHIMERE, EA7516, Université de Picardie Jules Verne, Amiens, France; 2 Laboratoire de Biochimie, Centre de Biologie Humaine, CHU Amiens, France; 3 Service d’Oncologie Médicale, CHU Amiens, France; 4 Service de Radiologie, CHU Amiens, France; 5 Laboratoire d’Oncobiologie Moléculaire, Centre de Biologie Humaine, CHU Amiens, France

**Keywords:** CCL2/MCP1, ERK1/2, Glioblastoma, Immune checkpoints, The Cancer Genome Atlas, Tumour-associated macrophages

## Abstract

The tumor microenvironment is an important determinant of glioblastoma (GBM) progression and response to treatment. How oncogenic signaling in GBM cells modulates the composition of the tumor microenvironment and its activation is unclear. We aimed to explore the potential local immunoregulatory function of ERK1/2 signaling in GBM. Using proteomic and transcriptomic data (RNA seq) available for GBM tumors from The Cancer Genome Atlas (TCGA), we show that GBM with high levels of phosphorylated ERK1/2 have increased infiltration of tumor-associated macrophages (TAM) with a non-inflammatory M2 polarization. Using three human GBM cell lines in culture, we confirmed the existence of ERK1/2-dependent regulation of the production of the macrophage chemoattractant CCL2/MCP1. In contrast with this positive regulation of TAM recruitment, we found no evidence of a direct effect of ERK1/2 signaling on two other important aspects of TAM regulation by GBM cells: (1) the expression of the immune checkpoint ligands PD-L1 and PD-L2, expressed at high mRNA levels in GBM compared with other solid tumors; (2) the production of the tumor metabolite lactate recently reported to dampen tumor immunity by interacting with the receptor GPR65 present on the surface of TAM. Taken together, our observations suggest that ERK1/2 signaling regulates the recruitment of TAM in the GBM microenvironment. These findings highlight some potentially important particularities of the immune microenvironment in GBM and could provide an explanation for the recent observation that GBM with activated ERK1/2 signaling may respond better to anti-PD1 therapeutics.

## Introduction

Glioblastoma (GBM) is the most common primary central nervous system malignancy in adults. It is characterized by its poor prognosis, with a median survival of <15 months from diagnosis [1]. The standard therapeutic approach involves primary surgery followed by temozolomide-based chemoradiotherapy [2]. The finding that GBM tumors exhibit frequent genomic alterations of receptor tyrosine kinases (RTK), such as the epidermal growth factor receptor (EGFR), raised hopes for therapeutic targetting in these tumors [3]. *In vitro* studies exploring the regulation and function of the ERK1/2 kinases, an essential downstream target of the RTK, have reported the role of ERK1/2 signaling in the regulation of GBM cell proliferation, invasion, apoptosis, tumor metabolism, and multiple other facets of tumor cell physiology [4]. Despite this strong rationale, Phase II/III clinical trials aiming to target RTKs, such as the EGFR, have been negative. This has led to skepticism regarding the interest of therapeutic strategies directed against oncogenic signaling in GBM [5].

Tumor immunotherapy based on the inhibition of immune checkpoints has conversely raised high expectations [6]. The first and the best characterized strategy used in clinics is based on the inhibition of the interaction of programmed cell death 1 (PD1) present on the surface of T lymphocytes with its ligands PD-L1/*CD274* and PD-L2/*PDCD1LG2* expressed by tumor cells and antigen presenting cells [6]. Biotherapies directed against PD1, such as nivolumab, were found to bring a significant benefit in the overall survival (OS) in malignant melanoma and other solid tumors by normalizing the tumor microenvironment [6]. The initial results for nivolumab, used as a single agent in recurrent GBM, have however failed to show a significant increase in the OS and objective responses were observed in less than 10% of patients (checkmate-143 study) [7]. It is currently unclear why a small fraction of GBM and other solid tumors respond better to PD1 targetting than others [8]. In addition to the expression of the ligands of PD1 (PD-L1 and PD-L2), other important parameters likely include the presence of immune cells within the tumor tissue, tumor mutational burden, genome repair ability, and the presence of a favorable cytokine context [8]. Single cell analyses suggest that malignant cells can instigate the formation of a ‘cold’ immune microenvironment and able to brake tumor-specific immune responses through active transcriptional mechanisms [9]. Whether oncogenic pathways contribute to these mechanisms is an interesting possibility that has to date been only superficially addressed [8]. Interestingly, a recent study by Zhao et al. identified frequent genomic alterations leading to ERK1/2 activation in a small subpopulation of GBM patients with objective responses to PD1 immunotherapy [10]. The present study opens up the possibility that ERK1/2 activation might be associated with specific properties of the local immune microenvironment in GBM, a possibility that has not yet been addressed.

Tumor-associated macrophages (TAM) represent a major component of the tumor microenvironment in GBM [11]. Several studies have suggested their contribution to the growth and aggressive behavior of GBM [11–14]. GBM cells can actively recruit TAM and promote their functional M2 polarization to a non-inflammatory phenotype, thereby preventing an efficient antitumor immune response [11]. In addition to the production of growth factors or cytokines, the functional polarization of TAM might be induced by metabolic products, such as lactate, produced by cancer cells as was recently shown in malignant melanoma [15,16]. Lactate, the main end product of glycolysis in cancer cells, is released in the tumor microenvironment where it is able to interact with specific receptors, such as the G-protein coupled receptor-65 (GPR65) [15,16]. In highly glycolytic tumors, such as malignant melanoma, Bohn et al. reported that lactate actively contributes to the noninflammatory M2 polarization of TAM via GPR65 activation [16]. GBM tumors are also known to use glycolysis [17]. Interestingly, metabolic regulation in general, and glycolysis in particular, are emerging as important effectors of ERK1/2 signaling in some solid tumors [18,19]. Whether the intermediary metabolism of tumor cells might regulate the immune microenvironment of GBM, possibly in an ERK1/2-dependent manner, has not yet been studied. In the present study, we aimed to address the regulatory role of ERK1/2 on the immune microenvironment of GBM. We retrieved multiomics data from The Cancer Genome Atlas (TCGA) to explore the GBM immune microenvironment [20,21]. We correlated the local immune characteristics of GBM tumors with ERK1/2 activation and validated our conclusions *in vitro* using human GBM cell lines.

## Materials and methods

### Patient data and tumor stratification

Basic clinical, pathological, and biological data were retrieved for a total number of 604 GBM tumors using cBioportal at: http://cbioportal.org [22,23] in September 2018. Reverse Phase Protein Array (RPPA) data were available for 244/604 GBM (*n*=40 phosphoproteins including phospho-ERK1/2, phospho-EGFR and phospho-MET). RNA Seq data (RNA SeqV2 data normalized using RNA-Seq by expectation maximization: RSEM) were available for 166/604 GBM. Overlapping RNAseq and RPPA data were available for *n=*82 tumors. Basic characteristics of the GBM patients in the RPPA cohort (*n=*238) are described in Table 1. Hierarchical clustering of the raw RPPA data was used to construct a dendrogram (complete, hclust R) in order to study the relatedness between the phosphoproteins and create a heatmap.

**Table 1:** GBM patient characteristics (*n=*238)

Age, years: median (range)	60 (18–88)
Gender	Male (*n=*143); female (*n=*94); NA (*n=*1)
Karnofsky Performance Score	<70 (*n=*51); 70–80 (*n=*101); 90 (*n=*4) 100 (*n=*25); NA (*n=*57)
Gene expression subtype*	Classical (*n=*53); mesenchymal (*n=*50) Proneural (*n=*43); neural (*n=*30) G-CIMP (*n=*14); NA (*n=*48)
IDH1 mutation status	WT (*n=*172); R132G (*n=*1); R132H (*n=*12) NA (*n=*53)
MGMT status	Methylated (*n=*69); unmethylated (*n=*82) NA (*n=*87)

NA: Not Available; G-CIMP: Glioma CpG island methylator phenotype; MGMT: O6-methylguanine-DNA methyl-transferase

* Defined according to Brennan et al. [3]

### Tumor immune infiltration analysis

The Microenvironment Cell Population-counter method was used to quantitate the relative abundance of eight types of immune and stromal cell populations based on RNA seq data [24]. Other immune parameters, such as M1/M2 polarization, were retrieved from Thorsson et al. [20].

### Cell culture

The human GBM cell lines A172, H4, and SW1088 were purchased from American Type Culture Collection (ATCC). The cell line A172 carries a tandem duplication of the *EGFR* gene [25]. The SK-MEL3 human melanoma cell line carries a heterozygous BRAF V600E mutation and was purchased from ATCC. All cell lines were cultured in DMEM supplemented with 10% fetal calf serum, 2 mM glutamine, penicillin, and streptomycin and maintained at 37°C in a humidified atmosphere with 5% CO_2_.

### Immunoblot analysis and ELISA

Rabbit antibodies directed against ERK1/2, ERK1/2 phosphorylated on Thr202/Tyr204 and EGFR were from Cell Signaling. Rabbit antibodies directed against PD-L1 and PD-L2 were from Abcam. Mouse antiactin was from Sigma. Proteins were precipitated, loaded on SDS/PAGE, and transferred to nitrocellulose membranes using standard procedures. ECL reaction was used for revelation [26]. Concentrations of the human cytokines CCL2/MCP1 (chemokine ligand 2/monocyte chemoattractant protein 1) and CSF1/MCSF (macrophage colony stimulating factor-1) were measured using ELISA kits, following the manufacturer’s instructions (both from R&D Systems, ref. DCP00 and DMC00).

### Quantitative PCR

Total RNA was extracted using TRIzol reagent and reverse-transcribed using Superscript VILO cDNA synthesis kit (ThermoFisher). Amplification was performed with the TaqMan Universal PCR master Mix on an ABI 7500 Real-Time PCR instrument (Applied Biosystems) using primers and probe sets specific for *CCL2/MCP1, CSF1/MCSF*, and *B2M* as reference (TaqMan Gene Expression Assay, Applied Biosystems).

### Lactate measurement

Lactate concentration was determined using a Siemens Vista analyzer and the adapted kit, approved for clinical use (Siemens).

### Statistical analyses

All analyses were done as indicated with *P*<0.05 as threshold of significance, using R version 3.4.2 (https://www.r-project.org).

## Results

### GBM classification and identification of a subgroup of GBM with high activation levels of ERK1/2

**Figure 1:**
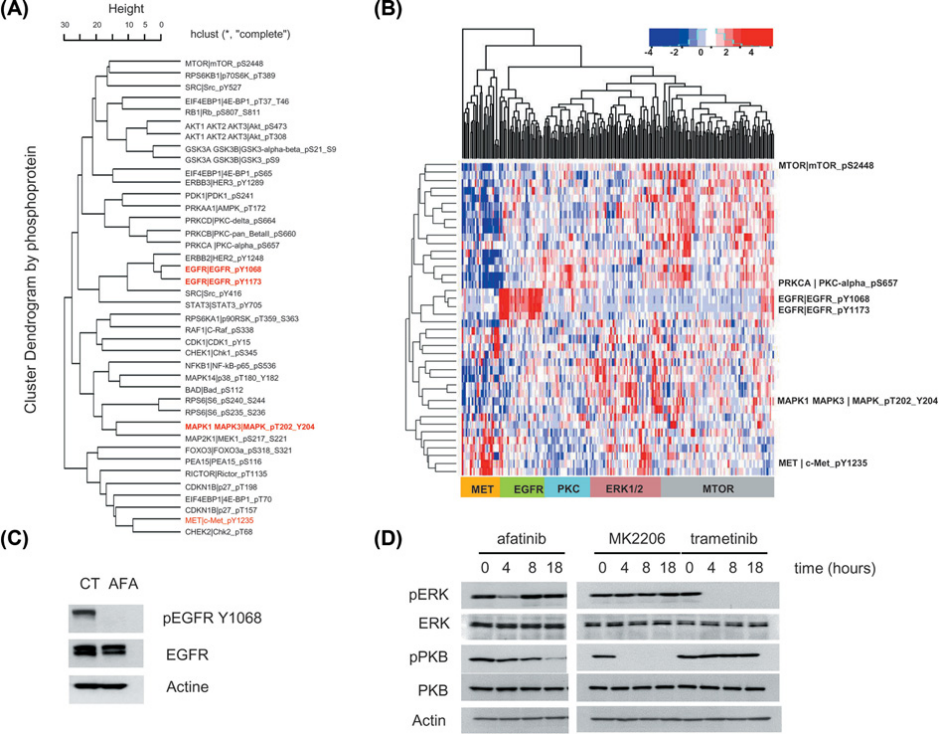
Identification of a subgroup of GBM with high phosphorylation levels of the ERK1/2 kinases (**A**) Hierarchical clustering of RPPA data (40 phosphoproteins including phosphorylated forms of ERK1/2, EGFR, and MET, highlighted in red) in GBM tumors from TCGA (*n=*244 samples). (**B**) Clustered heatmap of RPPA data for GBM with samples (columns) and phosphoproteins (rows). High protein expression is shown in red, low protein expression in blue. The following clusters are labeled at the bottom of the heatmap: MET, EGFR, PKC, ERK1/2 and MTOR. (**C**) Immunoblot analysis of EGFR phosphorylation in the human GBM cell line A172, carrying an amplified *EGFR* gene. Phosphorylation of EGFR on Y1068 (its major autophosphorylation site) reflects the activation of EGFR. Note the complete inhibition of phosphorylation upon exposure of A172 cells to afatinib (1 μM, 18 h). (**D**) A kinetic assessment of oncogenic signaling upon exposure of A172 cells to afatinib, MK2206 (2.5 μM, Akt/PKB inhibitor) or trametinib (1 μM, MEK inhibitor) both applied to A172 cells for the indicated time.

In order to address the role of ERK1/2 signaling in GBM tumors, we performed hierarchical clustering using RPPA data from TCGA. Clustering was based on *n=*244 GBM samples (238 patients, see Table 1) and *n=*40 phosphoproteins (including phosphorylated forms of ERK1/2, EGFR, and MET) (Figure 1A,B). Tumors with high levels of phospho-ERK1/2, phospho-EGFR, and phospho-MET were attributed to three separate clusters (Figure 1A,B). In order to provide an experimental support for the existence of this separate subgroup of GBM tumors characterized by high levels of phospho-ERK1/2, we used human GBM cell lines in culture. The GBM cell line A172, carrying a genomic amplification of EGFR [25] was exposed to selective chemical inhibitors of oncogenic signaling. Afatinib, a potent and selective anti-EGFR, radically blocked the phosphorylation of EGFR (Figure 1C). Afatinib, however, only slightly and temporarily inhibited ERK1/2 phosphorylation (Figure 1D). In contrast, the chemical inhibitors MK2206 and trametinib used as Akt/PKB and MEK1/2 inhibitors, respectively, dramatically and specifically blocked downstream signaling in GBM cells (Figure 1D). These findings suggested the existence of some form of functional uncoupling between EGFR and ERK1/2 in GBM cells and supported the existence of a distinct subset of GBM tumors characterized by high levels of phospho-ERK1/2.

### GBM tumors with high levels of phospho-ERK1/2 have a dense monocytic infiltrate

**Figure 2:**
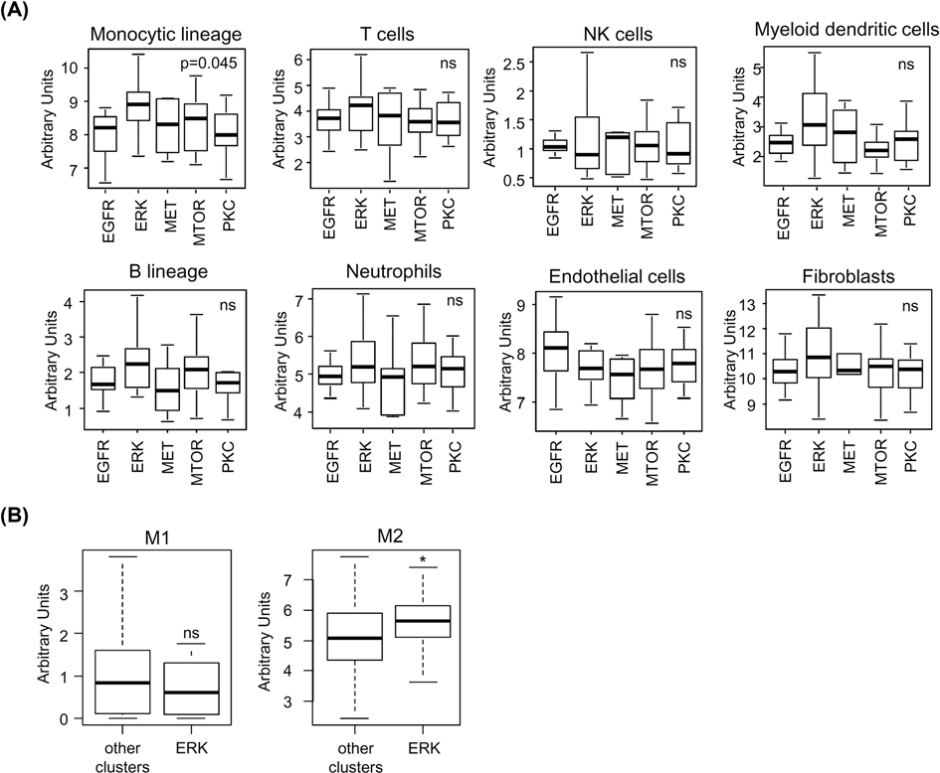
GBM tumors with high levels of phosphorylated ERK1/2 are characterized by a dense monocyte-macrophage infiltrate (**A**) Box plot analysis showing the relative levels of tumor infiltration for eight cell types based on transcript level analysis (MCP-counter), for tumors from the indicated GBM clusters: EGFR (*n=*12), ERK (*n=*18), MET (*n=*6), MTOR (*n=*23), and PKC (*n=*12). *P* values obtained with Kruskal–Wallis analysis; ns: nonsignificant. (**B**) M1/M2 polarization is based on the comparison of GBM tumors with high levels of ERK1/2 phosphorylation with all other GBM. *P<0.05

A pan-tumor comparison of GBM vs other major types of solid tumors in TCGA, based on transcriptomic analysis, showed that GBM present general characteristics of a « cold » immune microenvironment, with relatively low levels of the main types of immune cells within the tumor tissue (Supplementary Figure S1) [24]. Cells of the monocytic lineage constitute a notable exception because GBM was ranked fifth amongst the major tumor types for the relative content of these cells (Supplementary Figure S1). Using the MCP counter algorithm, we explored the relative content of eight immune and stromal cell types in the clusters that were previously identified (Figure 1A,B) We observed that GBM tumors with high levels of phospho-ERK1/2 were significantly enriched in cells of the monocytic lineage (*P*=0.045, Kruskal–Wallis) (Figure 2A). Other cell types were not found to be present at significantly higher density in the ERK1/2 subgroup, although a tendency toward higher infiltration levels was also observed for myeloid-dendritic cells (*P*=0.118, Kruskal–Wallis) (Figure 2A). Interestingly, analysis of macrophage polarization using transcriptomic data showed that GBM with higher phosphorylation of ERK1/2 were enriched in monocytes-macrophages with M2, but not M1 polarization (Figure 2B). We concluded that tumors with high phospho-ERK1/2 are characterized by their enrichment in TAM with a non-inflammatory M2 phenotype.

**Figure 3:**
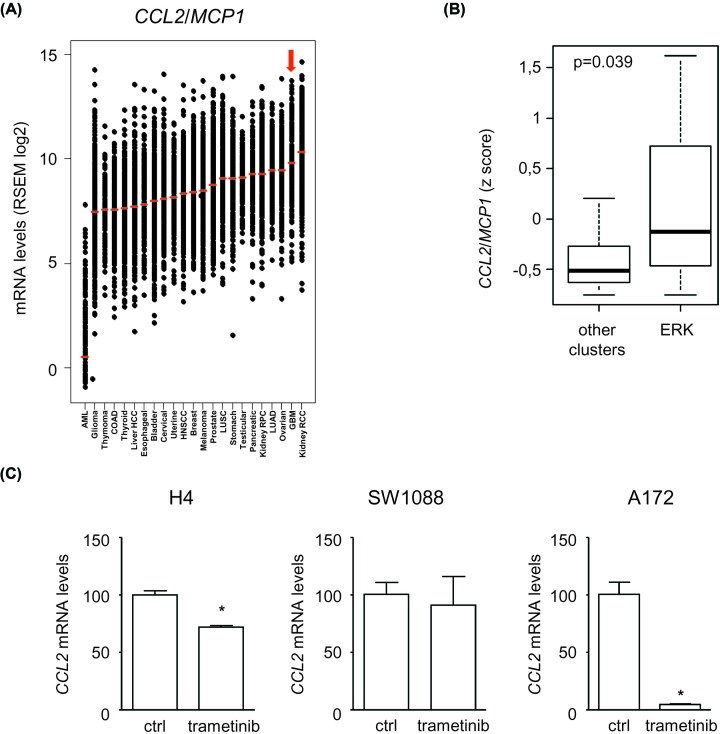
ERK1/2 signaling regulates CCL2/MCP1 expression in GBM (**A**) Tumor type ranking according to the expression levels of the *CCL2/MCP1* mRNA. Data were retrieved from TCGA, with *n=*23 tumor types and a total number of *n=*8823 tumors. GBM is indicated with the red arrow. (**B**) Box plot comparison of CCL2/MCP1 mRNA in GBM tumors with high ERK1/2 phosphorylation levels vs others (*P*=0.039 using Wilcoxon–Mann–Whitney test). (**C**) Quantitative PCR analysis of *CCL2/MCP1* mRNA levels in the indicated GBM cell lines (trametinib at 1 μM for 24 h). *P<0.05

**Figure 4:**
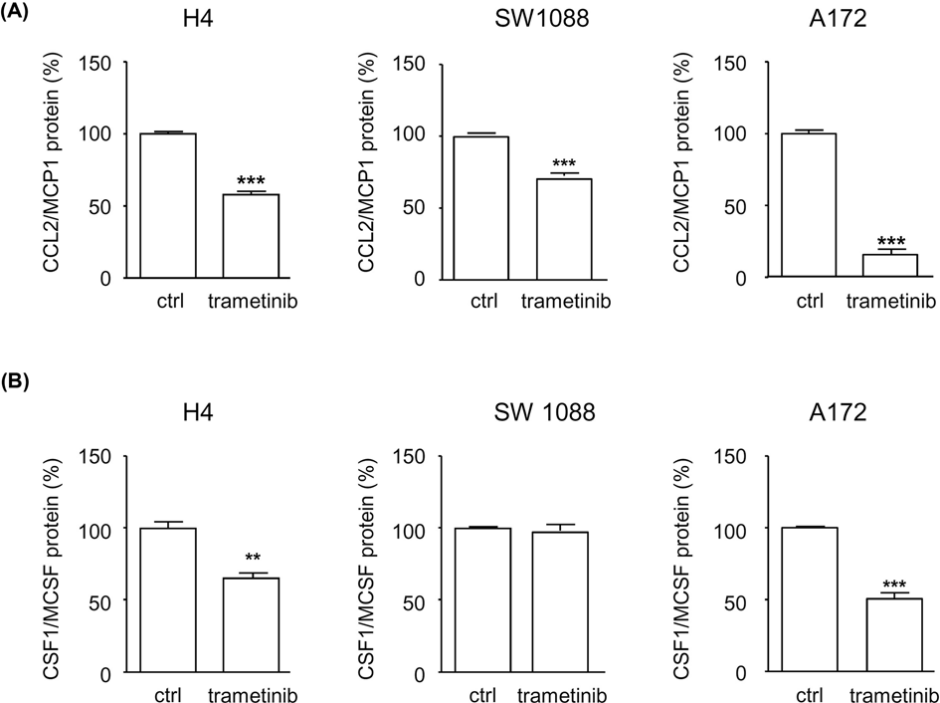
ERK1/2 signaling regulates the production of the cytokines CCL2/MCP1 and CSF1/MCSF in GBM (**A**) Protein concentration of CCL2/MCP1 and (**B**) CSF1/MCSF measured in the cell culture supernatant of GBM cell lines. Trametinib was applied as indicated at a concentration of 1 μM for 24 h. Each measurement was performed in triplicate. ***P<0.001 compared with control conditions, **P<0.01 (Student’s t test).

We examined the expression of the two main chemoattractants of monocytes: CCL2/MCP1 and CSF1/M-CSF. Using data from TCGA, we noticed that GBM is one of the solid tumor types that expresses the highest levels of the corresponding mRNA (Figure 3A, Supplementary Figure S2). GBM tumors within the ERK1/2 cluster expressed even higher levels of *CCL2/MCP1* mRNA compared with other clusters (*P*=0.039, Wilcoxon–Mann–Whitney) (Figure 3B). We addressed the regulation of the production of this chemokine experimentally in three human GBM cell lines (H4, SW1088, and A172) that were exposed to the MEK inhibitor trametinib, previously shown to block ERK1/2 signaling (Figure 1D). We showed that MEK inhibition significantly decreased CCL2/MCP1 mRNA levels in A172 cells (95% inhibition) and H4 cells (28% inhibition) (Figure 3C). Comparable results were obtained for CSF1 mRNA in GBM cells exposed to trametinib, as shown in Supplementary Figure S2. The protein levels of CCL2/MCP1 and CSF1/MCSF were measured in the cell culture supernatants using specific ELISA (Figure 4). We observed a strong decrease in the concentration of CCL2/MCP1 upon cell exposure to trametinib (an inhibition of 29–84% after adjustment to the number of cells, depending on the cell line, *P*<0.001 Student’s t test) (Figure 4A). A similar effect of trametinib was noted with CSF1, although to a less impressive extent, in two cell lines out of three (Figure 4B). MK2206 or afatinib, respectively, also reduced the protein levels of CCL2/MCP1 and CSF1/MCSF in the three GBM cell lines (Supplementary Figure S3). Importantly, however, in contrast with trametinib or MK2206, afatinib had a strong inhibitory effect on the viability of GBM cells upon prolonged *in vitro* exposure (data not shown). This observation lead us to question the relevance of the effect of afatanib on the production of CCL2 and CSF1, but at the same time, it highlighted the specificity of our observations with trametinib. We concluded that oncogenic signaling, especially via ERK1/2 directly regulates the ability of GBM cells to attract monocytes and macrophages.

### Lack of evidence for a direct control of PD-L1/PD-L2 expression or lactate metabolism by ERK1/2 signaling in GBM cells

**Figure 5:**
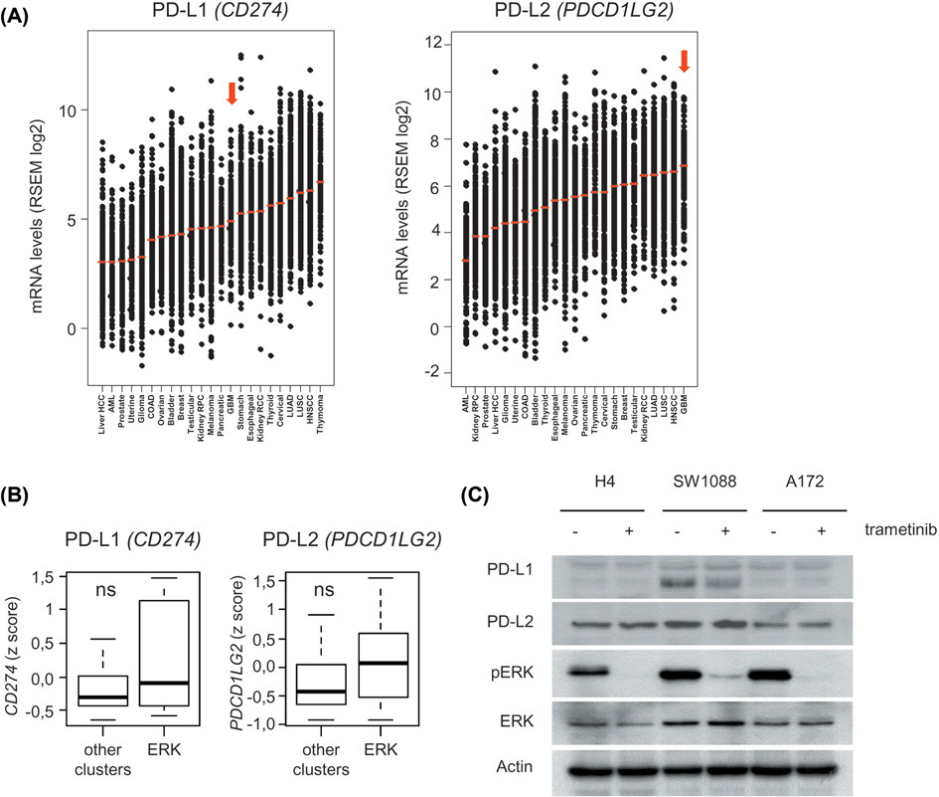
Lack of evidence of a direct regulation of the immune checkpoint ligands PD-L1 / PD-L2 by ERK1/2 signaling in GBM (**A**) Pan-cancer analysis of the expression of the immune checkpoint ligands PD-L1/PD-L2. Tumor types are ranked according to their mRNA expression levels of the immune checkpoint ligands PD-L1 (CD274) and PDL2 (PDCD1LG2). Data are from *n=*23 tumor types and a total number of *n=*8823 tumors. GBM is indicated with the red arrow. (**B**) Box plot comparison of PDL1 and PDL2 mRNA expression levels in GBM tumors with high ERK1/2 phosphorylation levels vs others (ns : nonsignificant; Wilcoxon–Mann–Whitney test). (**C**) Immunoblot analysis of PD-L1 and PD-L2 expression in GBM cell lines. Protein extracts were prepared from the indicated cell lines, exposed to trametinib (1 μM, 24 h) as indicated.

To further explore the potential link between ERK1/2 signaling and the regulation of TAM in GBM, we examined the expression of the mRNA encoding PD-L1/CD274 and PD-L2. A marked particularity of GBM was their high expression of PD-L2: a pan-cancer comparison showed that GBM express average levels of PD-L1 compared with other tumor types, but GBM is the tumor type with the highest relative expression of PD-L2 mRNA (Figure 5A). However, no significant difference in the expression of PD-L1 or PD-L2 mRNA was seen between the different GBM subgroups that we had previously identified (Figure 5B). The observation that GBM express high levels of PD-L2 was confirmed by immunoblot analysis (Figure 5C). We analyzed the human GBM cell lines H4, SW1088, and A172, and detected PD-L2 in all three cell lines, compared with PD-L1 that was found to be expressed at high levels in only one cell line (SW1088) (Figure 5C). Cell exposure to trametinib did not have a detectable effect on the expression of PD-L2, and a modest reduction in PD-L1 expression was detected in SW1088 cells (Figure 5C). Neither MK2206 nor afatinib altered the expression of PD-L1/PD-L2 by GBM cells in these conditions (Supplementary Figure S4).

**Figure 6:**
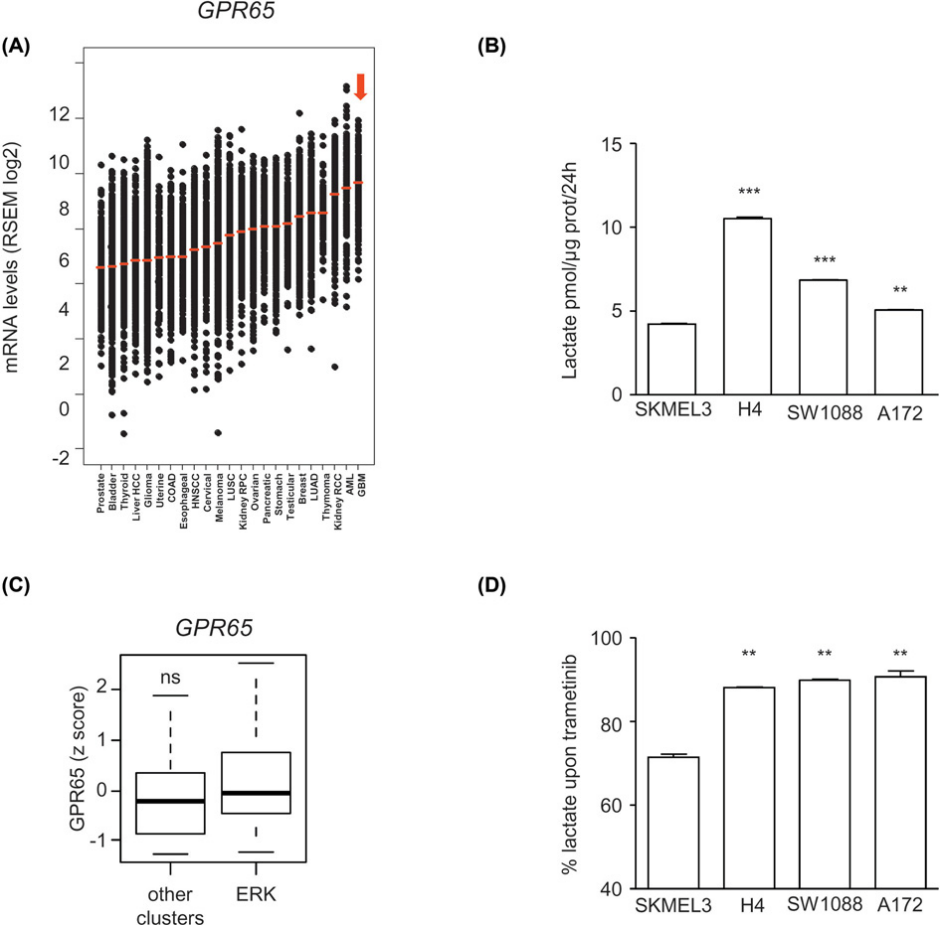
Lack of evidence of modulation of the metabolic inhibitory signaling mediated by lactate and its macrophage receptor GPR65 by ERK1/2 in GBM (**A**) Tumor ranking according to the expression levels of *GPR65* mRNA. Data are from *n=*23 tumor types and a total number of *n=*8823 tumors. GBM is indicated with the red arrow. (**B**) Lactate concentrations measured in the cell supernatant of GBM cells vs the melanoma cell line SK-MEL3. Lactate concentrations are expressed as pmol/μg prot/24 h. (**C**) Box plot comparison of GBM tumors with high levels of ERK1/2 phosphorylation vs other GBM (ns: not significant, Wilcoxon–Mann–Whitney test). (**D**) Inhibition of tumor glycolysis upon ERK1/2 inhibition by trametinib. Data are expressed as percent of lactate production upon exposure to trametinib (1 μM, 24 h), of the indicated GBM cell lines, taking control conditions as 100%. The cell line SK-MEL-3, carrying an activated BRAF V600E oncogene, is given here as positive control. Note the significantly greater inhibition of lactate production in SK-MEL-3 compared with GBM cells. **P<0.01, ***P<0.001 compared with SK-MEL3.

In order to address the existence of metabolic regulation of the tumor immune microenvironment, we analyzed the expression levels of the lactate receptor GPR65, a cell surface receptor recently reported to dampen local tumor immunity by interfering with the function of TAM [15,16]. GBM was found to be the tumor type with the highest expression levels of the lactate receptor GPR65 at the mRNA level (Figure 6A). Lactate was also detected at high concentrations in the cell culture supernatant of GBM cells; in order to compare GBM with the model situation analyzed by Bohn et al. [16], we compared the flux of lactate produced by GBM cells with the malignant melanoma cell line SK-MEL3 (BRAF V600E). Interestingly, GBM systematically produced more lactate *in vitro* than SK-MEL3 cells: a relative difference of 2.5-fold was observed for H4 cells vs SK-MEL3 : 10.5 vs 4.2 pmol lactate/μg protein/24 h, respectively (*P*<0.001, Student’s t test) (Figure 6B). These findings suggest that lactate signaling is active in the GBM tumor microenvironment. However, we found no indication that this aspect was regulated by ERK1/2 signaling. GBM tumors with high levels of ERK1/2 phosphorylation did not have significantly higher levels of GPR65 (Figure 6C). Similarly, lactate production by GBM cells was only modestly inhibited by trametinib (<10% inhibition for all three GBM cell lines tested, compared with a 30% inhibition in SK-MEL3, *P*<0.001, Student’s t test) (Figure 6D). In comparison, lactate production by GBM cells was strongly inhibited by MK2206 (Supplementary Figure S5). We concluded that ERK1/2 signaling is not directly involved in the lactate-related control of the GBM tumor microenvironment.

## Discussion

The present study aimed to explore the local immunoregulatory function of ERK1/2 signaling in GBM cells. We based our analysis on the comparison of GBM vs other solid tumors, and we also analyzed a subset of GBM with high phosphorylation of ERK1/2. Our study led us to address the regulation of TAM, a major component of the immune infiltrate in GBM [11]. Interestingly, the presence of TAM has been reported to be an important determinant of GBM aggressiveness and to potentially play a role in the response of GBM to various therapies [11–14]. We found that TAM infiltration was denser in tumors with high levels of ERK1/2 phosphorylation compared with others. We found that GBM produce large amounts of the monocyte chemoattractant cytokines CCL2/MCP1 and CSF1/MCSF, and the inhibition of ERK1/2 strongly reduced this production. To our knowledge, the present study is the first to directly show that ERK1/2 signaling in GBM cells can shape the tumor immune microenvironment. Nevertheless, our conclusions are supported by a previous report examining the impact of *NF1* gene deficiency in GBM [14]. NF1 deficiency, thought to reduce RAS GTPase activity and activate ERK1/2, was reported to increase TAM infiltration in GBM tumors [14]. These findings collectively highlight the contribution of ERK1/2 signaling in GBM cells in the recruitment of TAM, an essential feature of the immune microenvironment of GBM.

Whether our findings explain why GBM carrying genomic alterations that activate ERK1/2 respond better to anti-PD1 immunotherapy is an interesting possibility that will require further experimental validation [10]. Cancer microenvironment is the product of multiple layers of regulation that converge to produce a setting favorable for tumor progression [27]. Our study was centered on the role of oncogenic signaling in GBM cells and did not consider ERK1/2 signaling in immune cells and TAM in particular [13,28]. In order to precisely define the end result of ERK1/2 inhibition in GBM, *in vivo* experiments will be required. However, *in vivo* targetting of ERK1/2 signaling in GBM remains challenging. In agreement with studies that point to the complex regulation of oncogenic signaling in GBM [29], we observed that EGFR targetting fails to robustly inhibit ERK1/2 signaling, even in a context of genomic amplification of this receptor. Robust inhibition of ERK1/2 signaling was achieved with trametinib, a chemical inhibitor that does not however have an optimal pharmacological profile for clinical use against brain tumors [30].

In addition to our conclusions regarding the recruitment of TAM in GBM, we explored the impact of ERK1/2 signaling on two mechanisms that regulate the activation of TAM in solid tumors [7]: (1) high expression of PD1 ligands (PD-L1 and PD-L2) in GBM cells and (2) the secretion of lactate combined with high expression of the gene encoding its receptor GPR65, recently found to be an essential contributor to the noninflammatory polarization of TAM [16]. Targetting oncogenic signaling to improve antitumor immunity is currently an attractive, yet speculative possibility. Recent studies suggest that in melanoma and breast carcinoma, tumor metabolism may be a key target of oncogenic signaling dowstream of ERK1/2 [18,19]. We found however no evidence of a direct control of ERK1/2 signaling over this potentially important aspect of TAM regulation in GBM. Our study therefore suggests that GBM cells exert a negative control over TAM independently of ERK1/2 signaling. Understanding the multiple facets of the regulation of the tumor microenvironment will be essential for successful immunotherapeutic treatment in GBM.

## Supporting information

**Supplementary Figure S1:** Analysis of the density of tumour infiltration with various types of immune and stromal cells

**Supplementary Figure S2:** CSF1 mRNA expression in GBM.

**Supplementary Figure S3:** Effect of afatinib and MK2206 on the production on CCL2/MCP1 and CSF1 in GBM

**Supplementary Figure S4:** Immunoblot analysis of the expression of the immune checkpoint ligands PD-L1/PD-L2 upon exposure to afatinib or MK2206 in GBM cells.

**Supplementary Figure S5:** Lactate production by GBM cells exposed to MK2206

## Abbreviations

ATCC: American Type Culture Collection
CCL2/MCP1: chemokine ligand 2/monocyte chemoattractant protein 1
CSF1/MCSF: macrophage colony stimulating factor-1
EGFR: epidermal growth factor receptor
GBM: glioblastoma
RPPA: reverse phase protein array
RTK: receptor tyrosine kinase
TAM: tumor-associated macrophage
TCGA: The Cancer Genome Atlas
