# Supplementary material for: ERK1/2 signaling regulates the immune microenvironment and macrophage recruitment in glioblastoma
Source: Biosci Rep. 2019 Sep 13;39(9):BSR20191433. doi: 10.1042/BSR20191433 (PMC6744584; doi:10.1042/BSR20191433)
Supplement: Supplementary file 1 [file bsr20191433_Supp1.pdf]

Claire Lailler *et al.*

ERK1/2 signaling regulates the immune microenvironment and macrophage recruitment in Glioblastoma

Suppl. Tables and Figures

Suppl. fig. 1

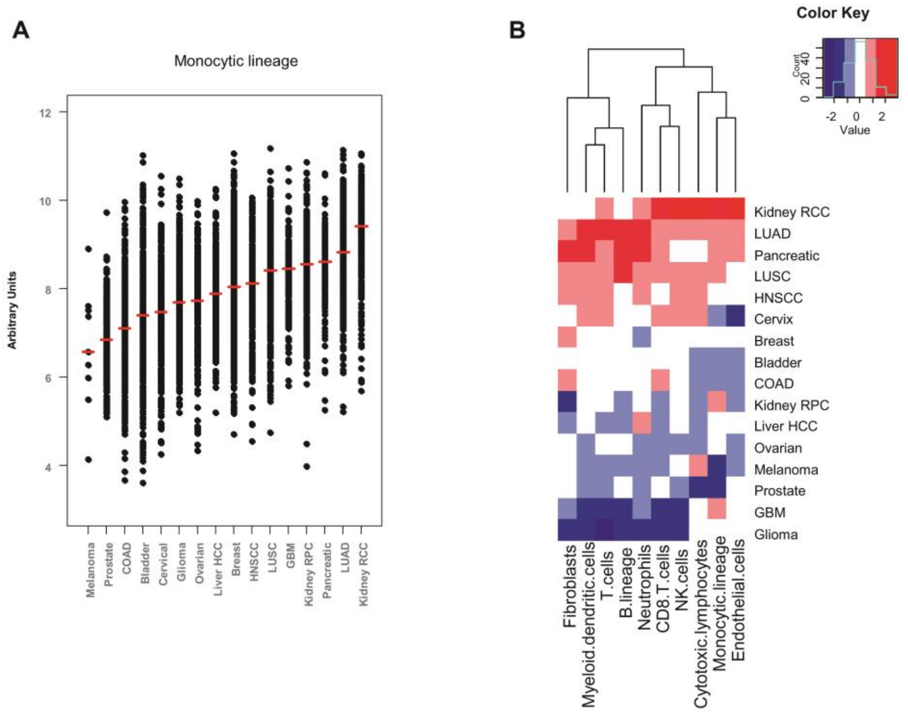

Suppl. Fig. 1: Analysis of the density of tumour infiltration with various types of immune and stromal cells.

A. A comparison of the monocyte-macrophage infiltrate of n=16 types of tumours from TCGA, based on the MCP counter analysis. Note that GBM is ranked as the 5th highest among all types of tumours examined in terms of monocyte infiltrate. B. Heatmap comparing the immune cell infiltrate in the 16 most common tumour types. Red corresponds to high immune/stromal infiltration, blue corresponds to « cold » tumours with low immune infiltration.

Suppl. fig. 2

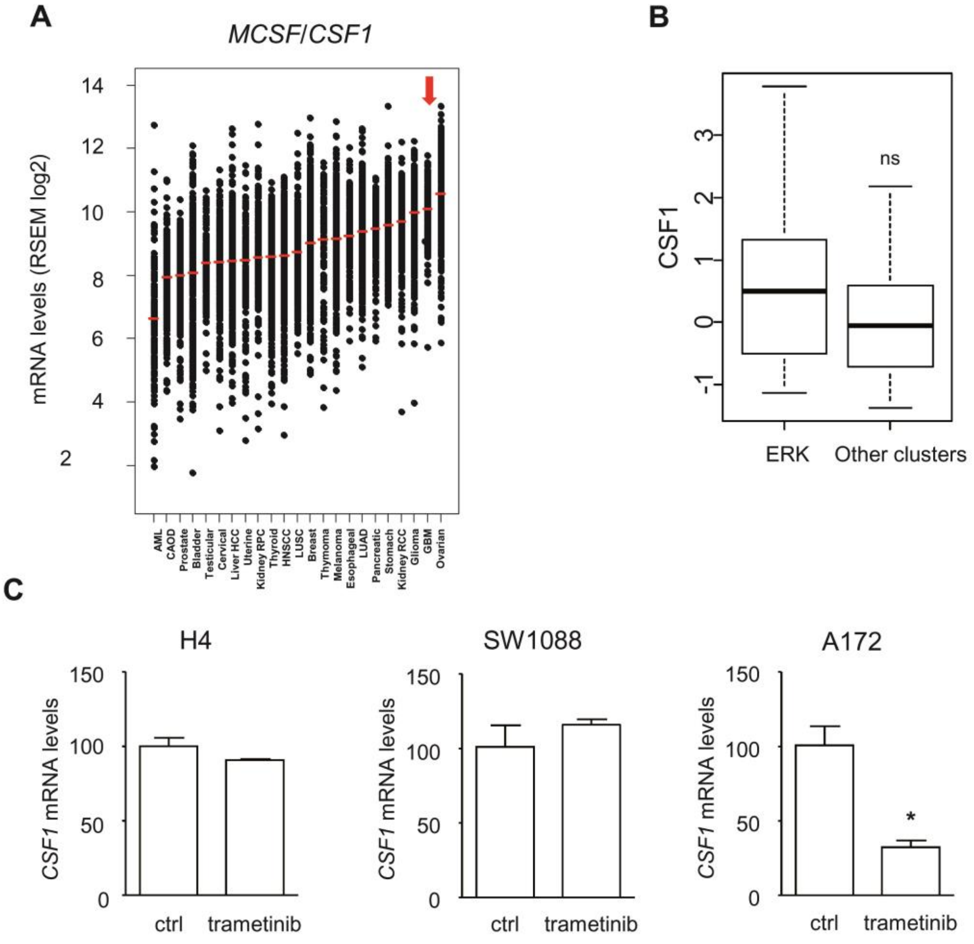

Suppl. Fig. 2: CSF1 mRNA expression in GBM.

A. Tumour type ranking according to the mRNA expression levels of CSF1. Data were retrieved from TCGA, with n=23 tumour types and a total number of n=8,823 tumours. Note that GBM are indicated with a red arrow. B. Box plot comparing GBM tumours with high levels of phosphorylation of ERK vs others (p=0.08 using Wilcoxon-Mann-Whitney test). C. Quantitative PCR analysis of CSF1 mRNA levels in the indicated GBM cell lines (trametinib 1 µM, 24h exposure).

Suppl. fig. 3

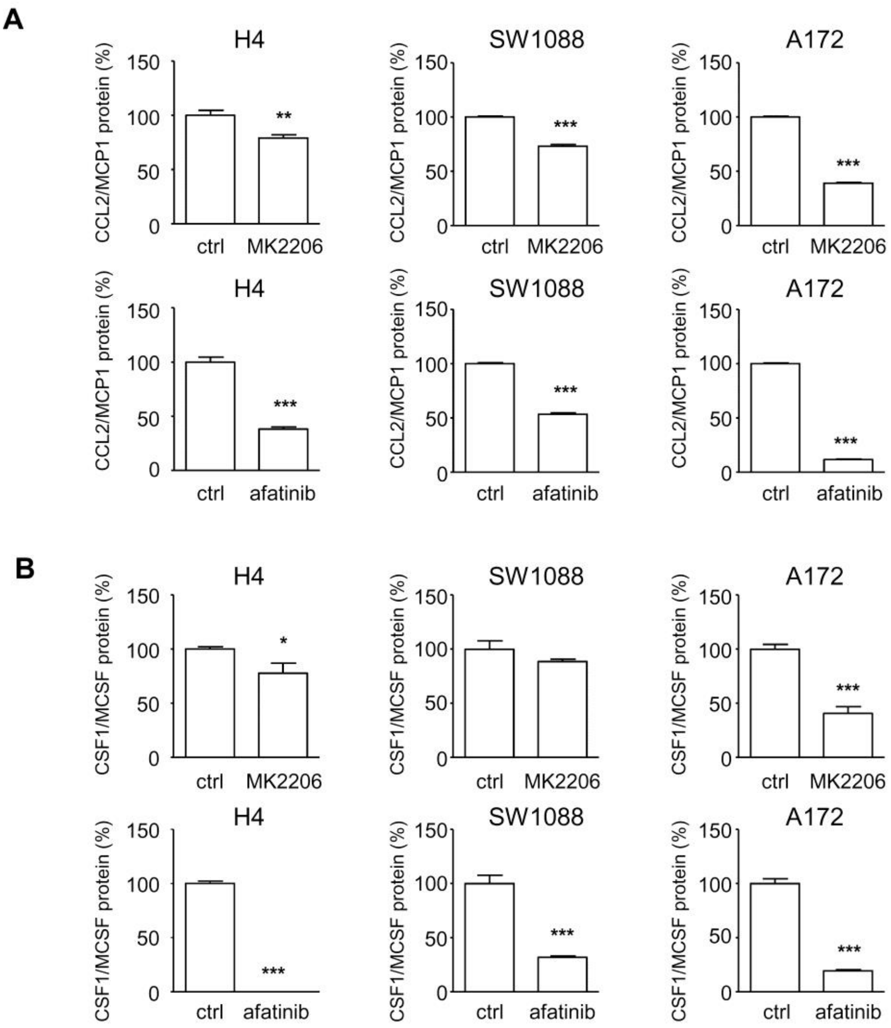

Suppl. Fig. 3: Effect of afatinib and MK2206 on the production on CCL2/MCP1 and CSF1 in GBM.

Protein concentration of CCL2/MCP1 and CSF1 were measured in the culture supernatants of GBM cell lines, with MK2206 and afatinib applied for 24h at a concentration of 2.5 and 1 µM, respectively. Each measurement was performed in triplicate. *** $p<0.001$ compared to control conditions, ** $p<0.01$, and * $p<0.05$ (Student's t test).

Suppl. fig. 4

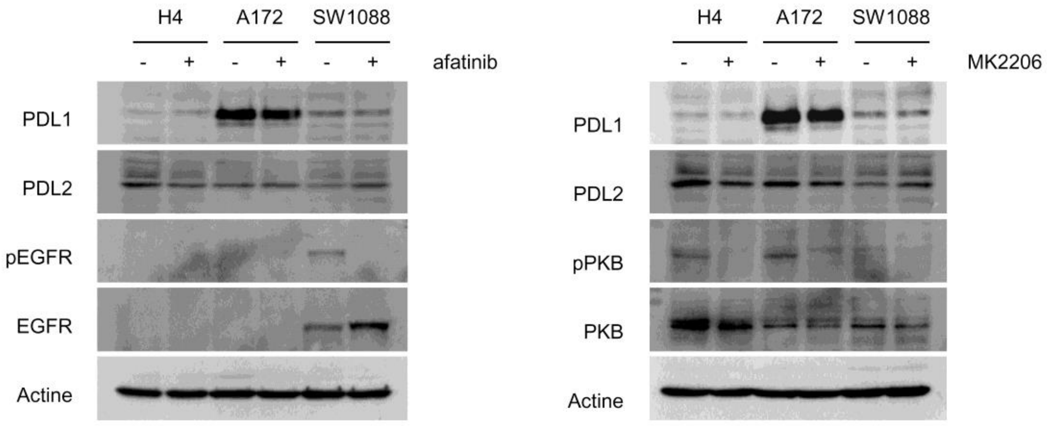

Suppl. Fig. 4: Immunoblot analysis of the expression of the immune checkpoint ligands PD-L1 / PD-L2 upon exposure to afatinib or MK2206 in GBM cells.

Immunoblot analysis of PD-L1 and PD-L2 expression in GBM cell lines. Protein extracts were prepared from the indicated cell lines, exposed to afatinib (1 µM) or MK2206 (2.5 µM) for 24h, as indicated.

Suppl. fig. 5

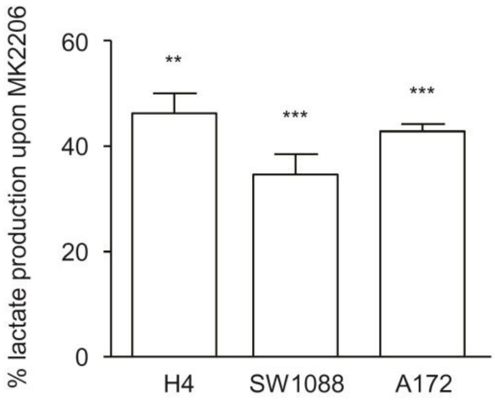

Suppl. Fig 5: Lactate production by GBM cells exposed to MK2206

Data are expressed as % lactate production by GBM upon exposure to MK2206 (2.5 µM). For each of the indicated GBM cell lines, the effect of MK2206 is expressed as % of lactate production upon exposure to MK2206, with 100% indicating the lactate production under control conditions. *** $p<0.001$, ** $p<0.01$ compared to control conditions.
